# Supplementary figures and images for: Discovering functional sequences with RELICS, an analysis method for CRISPR screens
Source: PLoS Comput Biol. 2020 Sep 16;16(9):e1008194. doi: 10.1371/journal.pcbi.1008194 (PMC7521704; doi:10.1371/journal.pcbi.1008194)

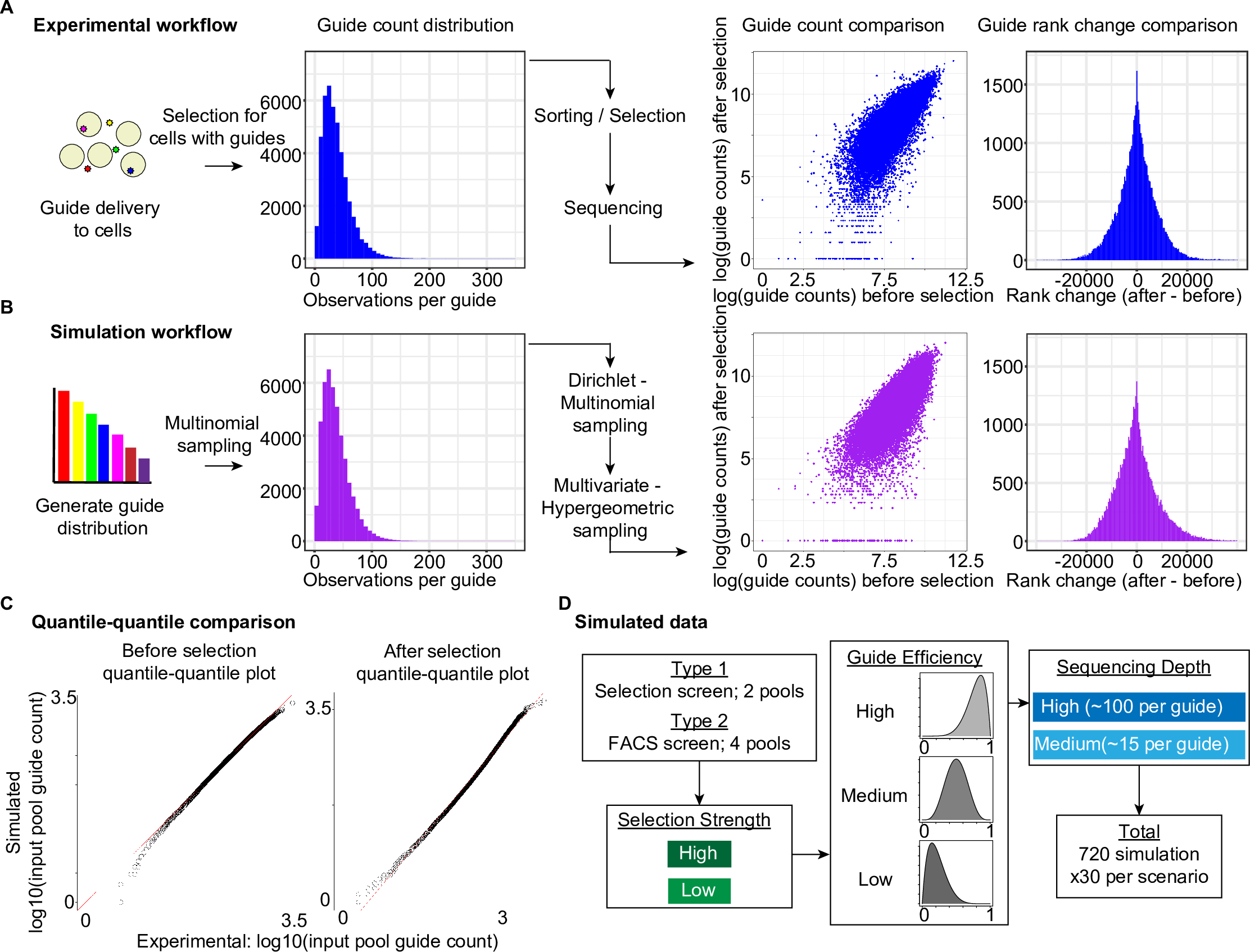

Supplement: S1 Fig — CRISPR screen simulation framework (A) Experimental workflow. sgRNAs are introduced into cells and only cells which receive sgRNAs are retained. These cells are then either sorted based on gene expression, or placed under selective pressure (e.g. for survival or proliferation). sgRNA counts and guide ranks before and after selection are shown. (B) In our simulations, we generate an initial sgRNA count distribution and mimic the experimental steps using several different sampling procedures. The simulated sgRNA counts for before and after simulated selection are shown for comparison with the experimental data. (C) Quantile-quantile plots of simulated vs experimental data, before and after selection. (D) Combinations of parameters used for the simulations. We simulated two different types of screens with two different selection strengths, 3 different guide efficiency distributions, and two sequencing depths. For each scenario, we simulated 30 data sets. (TIF) [file pcbi.1008194.s001.tif]

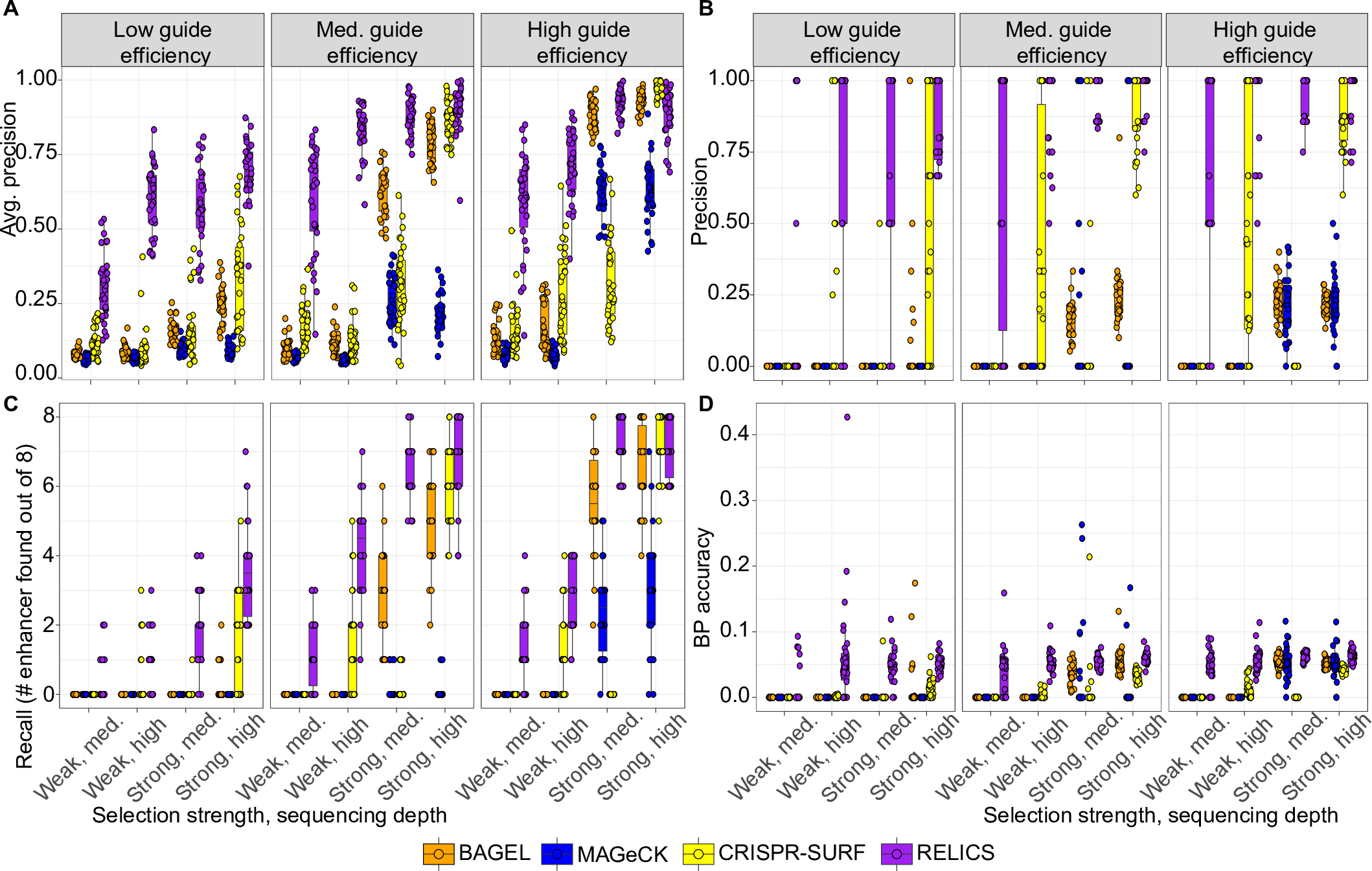

Supplement: S2 Fig — (A) Boxplots of average precision for different simulation parameters. (B) Boxplots of precision, the fraction of significant regions that contain a simulated enhancer. (C) Boxplots of recall, the number of simulated enhancers detected (out of 8) amongst the significant regions identified. (D) Boxplots of base pair (BP) accuracy, the fraction of base pairs in significant regions that overlap a simulated enhancer. The hinges of the boxplots correspond to the first and third quartiles, the center lines are the medians, and the whiskers extend to the furthest datapoints that are within 1.5x the interquartile range from the hinge. (TIF) [file pcbi.1008194.s002.tif]

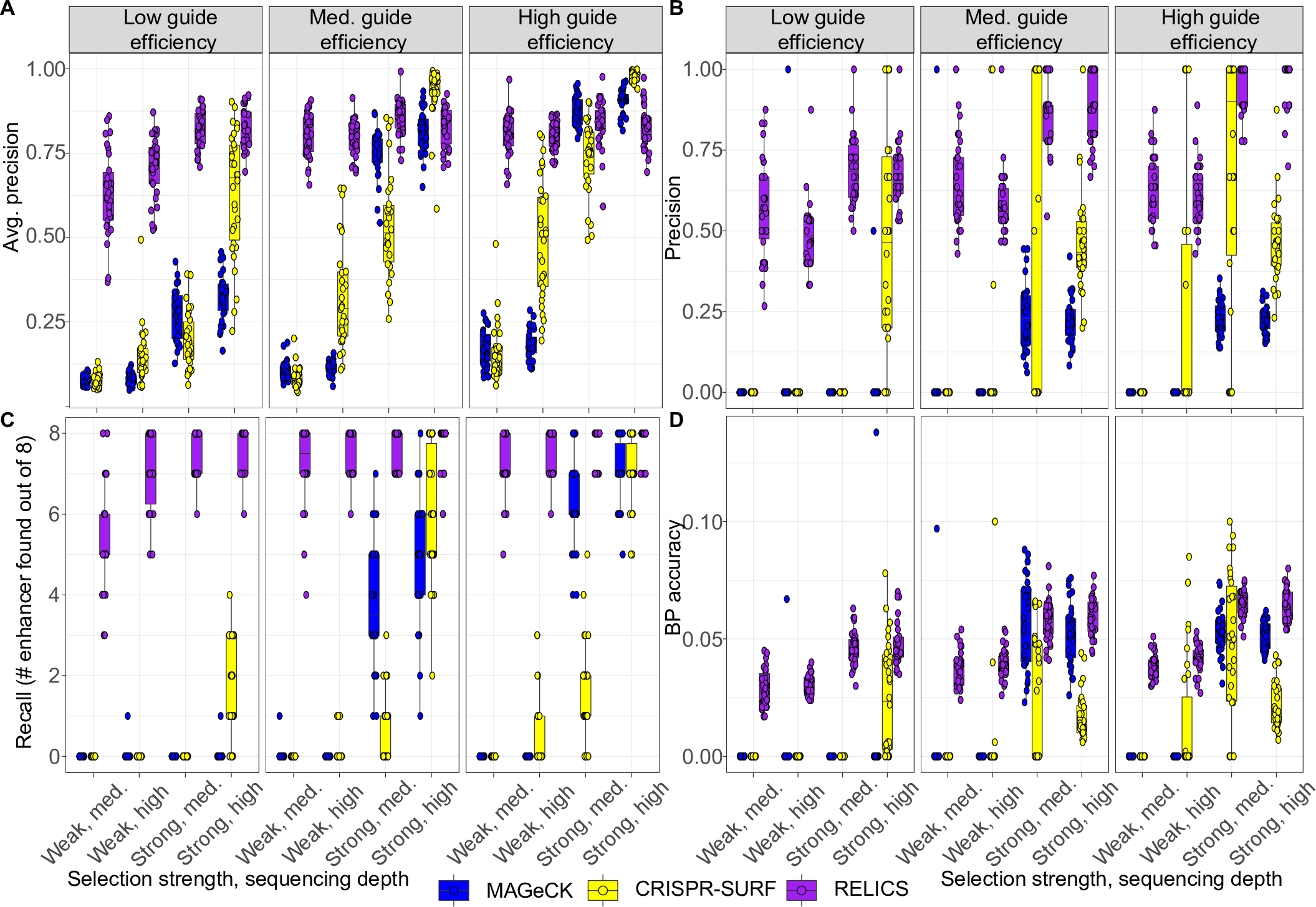

Supplement: S3 Fig — (A) Boxplots of average precision for different simulation parameters. (B) Boxplots of precision, the fraction of significant regions that contain a simulated enhancer. (C) Boxplots of recall, the number of simulated enhancers detected (out of 8) amongst the significant regions identified. (D) Boxplots of base pair (BP) accuracy, the fraction of base pairs in significant regions that overlap a simulated enhancer. The hinges of the boxplots correspond to the first and third quartiles, the center lines are the medians, and the whiskers extend to the furthest datapoints that are within 1.5x the interquartile range from the hinge. (TIF) [file pcbi.1008194.s003.tif]

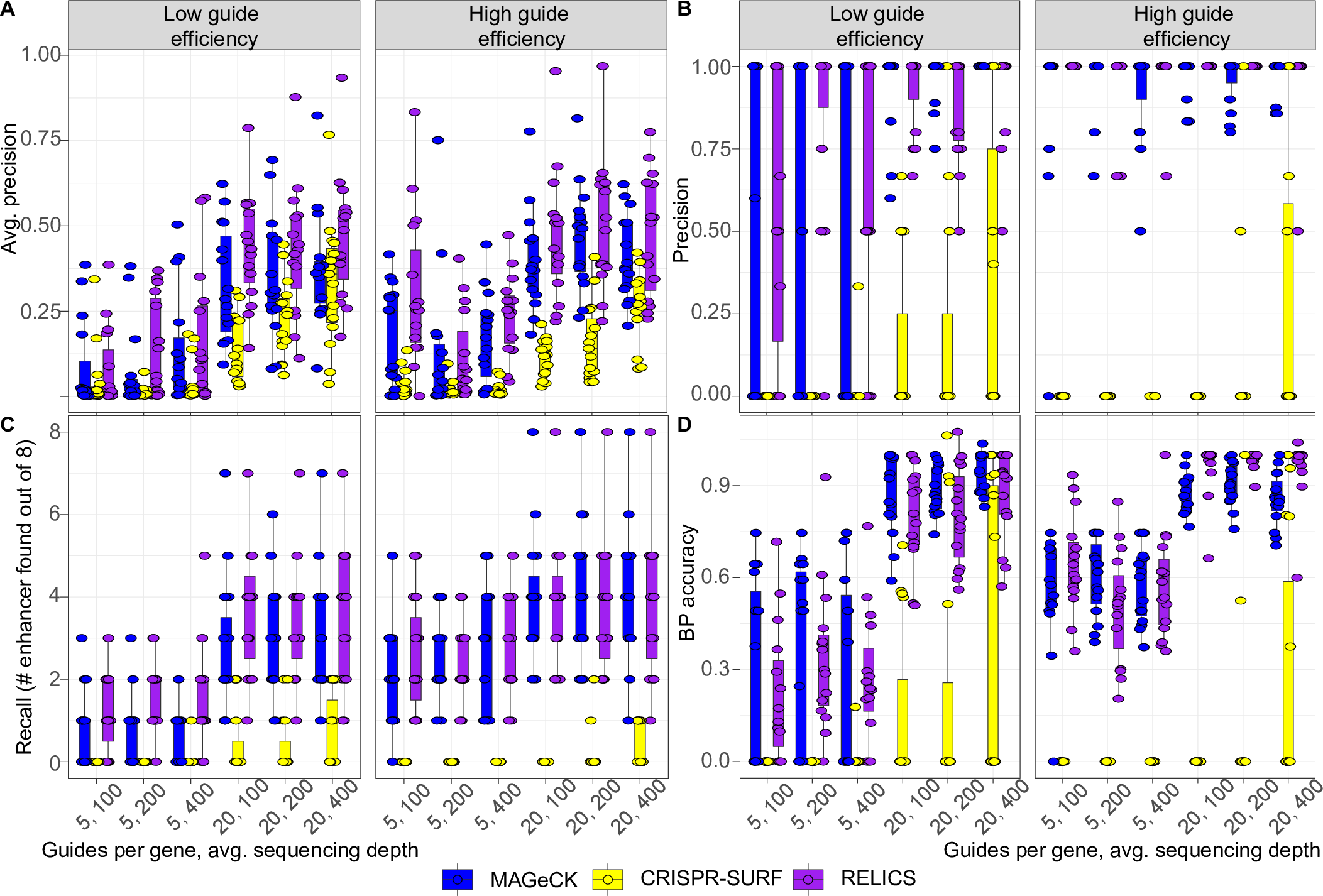

Supplement: S4 Fig — The simulated data is for a selection screen with 2 pools. (A) Boxplots of average precision for different simulation parameters. (B) Boxplots of precision, the fraction of significant regions that contain a simulated enhancer. (C) Boxplots of recall, the number of simulated enhancers detected (out of 8) amongst the significant regions identified. (D) Boxplots of base pair (BP) accuracy, the fraction of base pairs in significant regions that overlap a simulated enhancer. The hinges of the boxplots correspond to the first and third quartiles, the center lines are the medians, and the whiskers extend to the furthest datapoints that are within 1.5x the interquartile range from the hinge. The simulation details are described in the methods. In 117/180 of the simulations RELICS had the highest AP. RELICS also had the highest precision (157/180), recall (163/180) and BP accuracy (102/180). (TIF) [file pcbi.1008194.s004.tif]

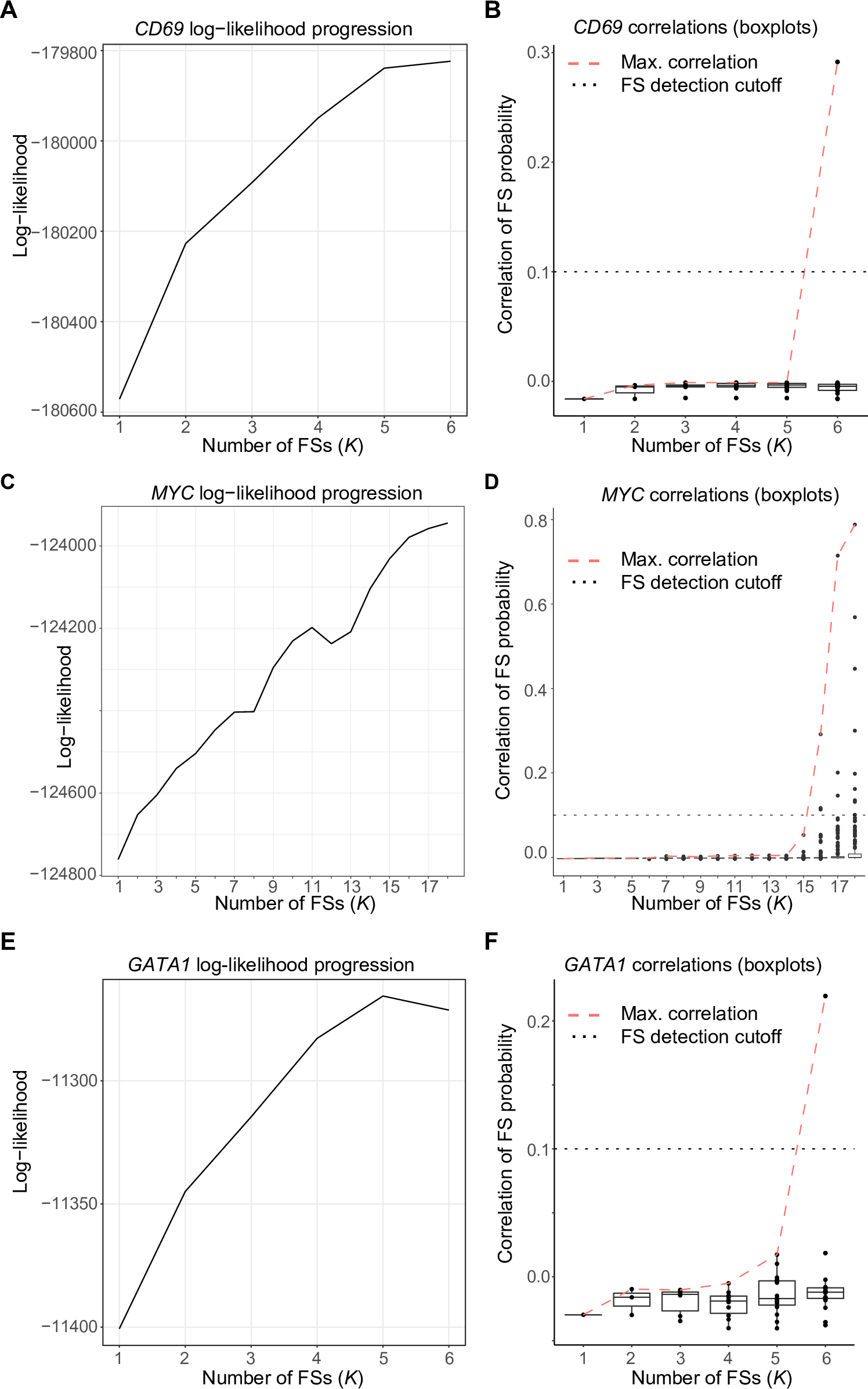

Supplement: S5 Fig — (A,C,E) Log likelihood as the number of FSs increases for CD69, MYC and GATA1. (B,D,F) Pairwise FS placement probability correlations for CD69, MYC and GATA1. The red dashed lines indicate the highest pairwise correlation across all FSs. The hinges of the boxplots in (B), (D) and (F) correspond to the first and third quartiles, the center lines are the medians, and the whiskers extend to the furthest datapoints that are within 1.5x the interquartile range from the hinge. (TIF) [file pcbi.1008194.s005.tif]

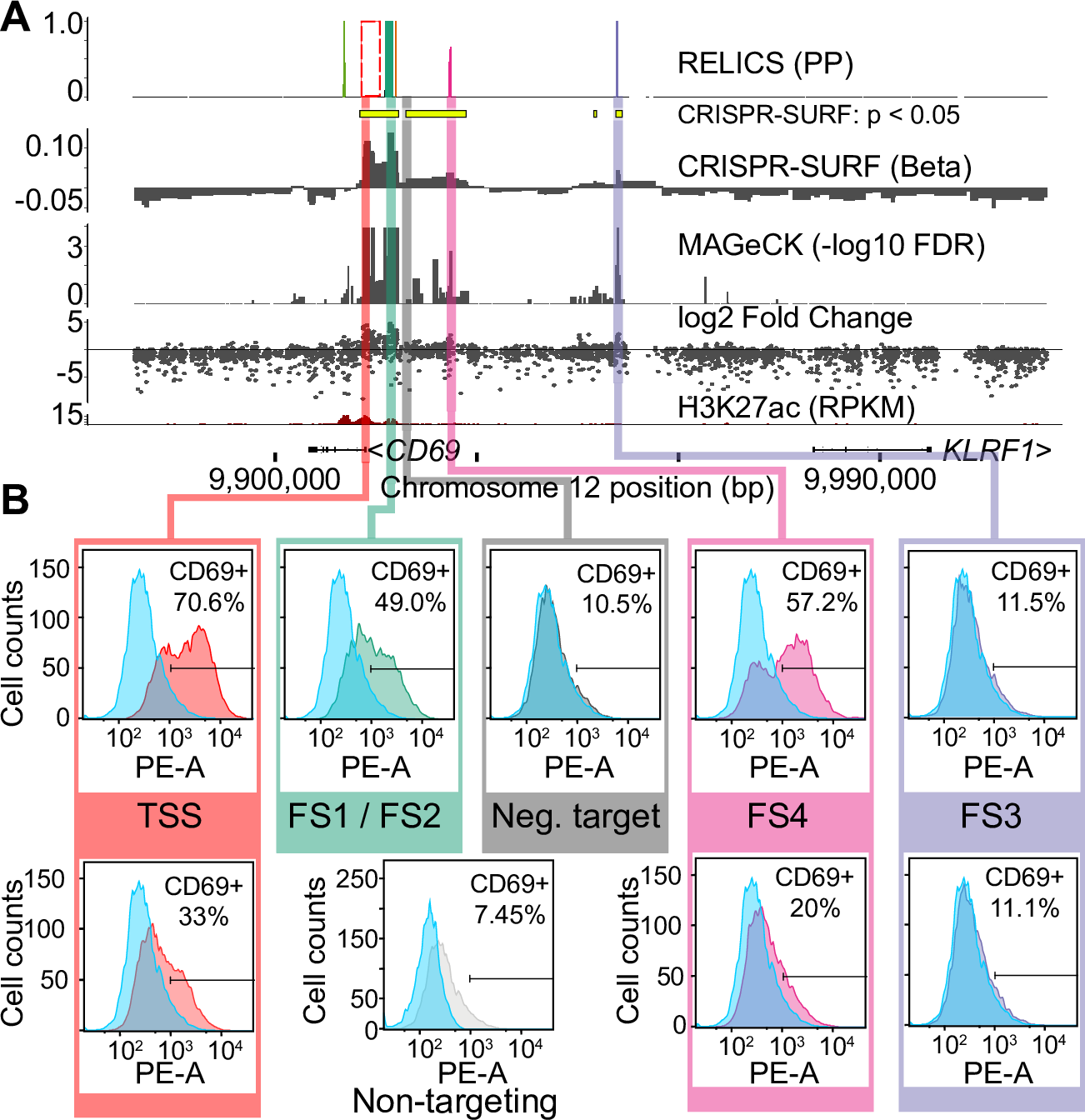

Supplement: S6 Fig — (A) Analysis results for the CD69 screen by RELICS, CRISPR-SURF, MAGeCK and log2 fold change. The RELICS probabilities for each FS are collapsed into a single track. An H3K27ac ChIP-seq track for Jurkat cells is included. The locations of sequences chosen for experimental validation are indicated by colored lines. (B) Results from validation experiments. Lentiviral vectors encoding sgRNAs and dCas9:VP64 were co-transduced into Jurkat cells, and the expression of CD69 protein was quantified by flow cytometry. Non-targeting sgRNAs were used as a negative control. Targeting sgRNAs were chosen for their specificity and high predicted efficiency (relative to other possible sgRNAs in the region) and in some cases are adjacent to the predicted FS rather than within the FS. (TIF) [file pcbi.1008194.s006.tif]

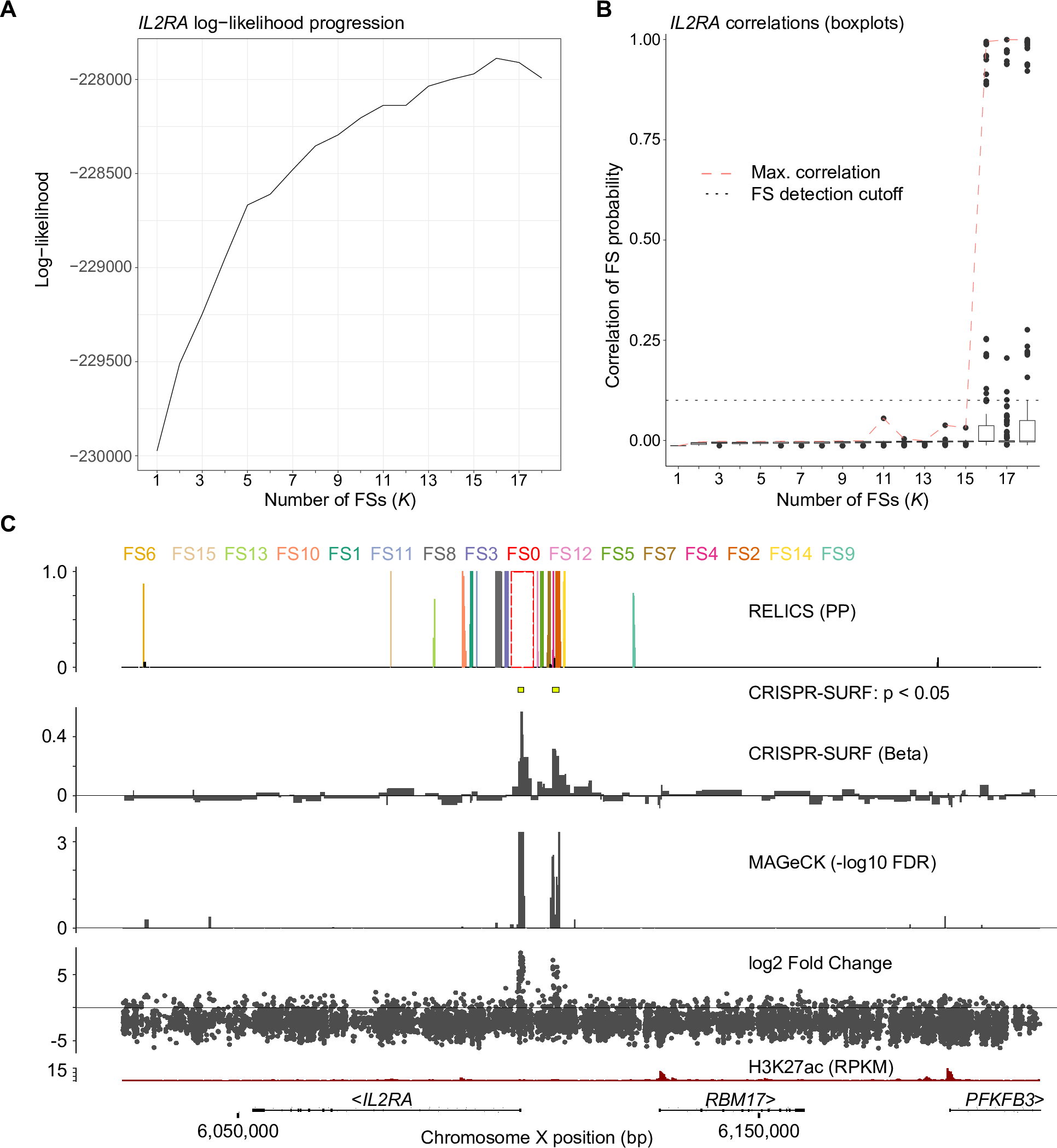

Supplement: S7 Fig — (A) Log-likelihood progression. With the addition of each functional sequence (FS), the model log-likelihood is recorded. (B) The pairwise correlations between functional sequence probabilities as a function of the number of FSs. The red dashed line indicates the highest pairwise correlation between all pairs of FSs. The hinges of the boxplots correspond to the first and third quartiles, the center lines are the medians, and the whiskers extend to the furthest datapoints that are within 1.5x the interquartile range from the hinge. (C) Output of RELICS and another analysis methods. Each FS predicted by RELICS is given a different color and the labels are arranged by genomic position. (TIF) [file pcbi.1008194.s007.tif]

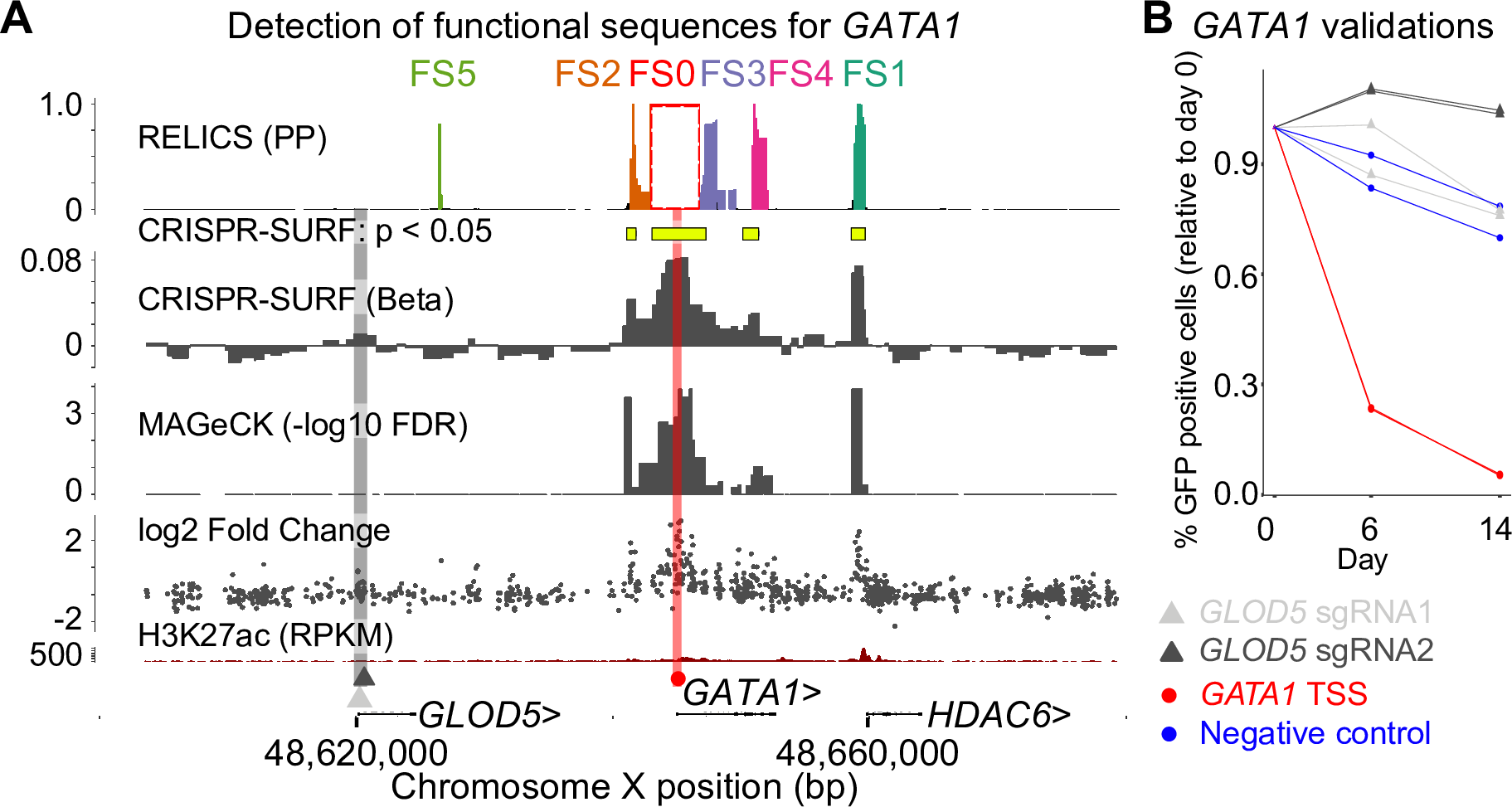

Supplement: S8 Fig — (A) Results from RELICS and other analysis methods. RELICS detects 5 functional sequences (FS1-5). FS1 and FS2 have previously been validated; FS3 and FS4 fall within GATA1. sgRNA target sites for validation experiments are indicated with a red circle (GATA1 promoter) and grey and black triangles (GLOD5). (B) Results from validation experiments (2 replicates) using sgRNAs targeting sites indicated in panel A. Each validation experiment is a cellular proliferation assay, in which the percent of GFP-positive cells (i.e. those that received the sgRNA) are measured at day 0, day 6 and day 14. While targeting the GATA1 promoter greatly reduces proliferation, targeting the GLOD5 region does not change proliferation compared to a negative control sgRNA, which targets a non-functional ‘safe harbor’ region on chromosome 8. (TIF) [file pcbi.1008194.s008.tif]

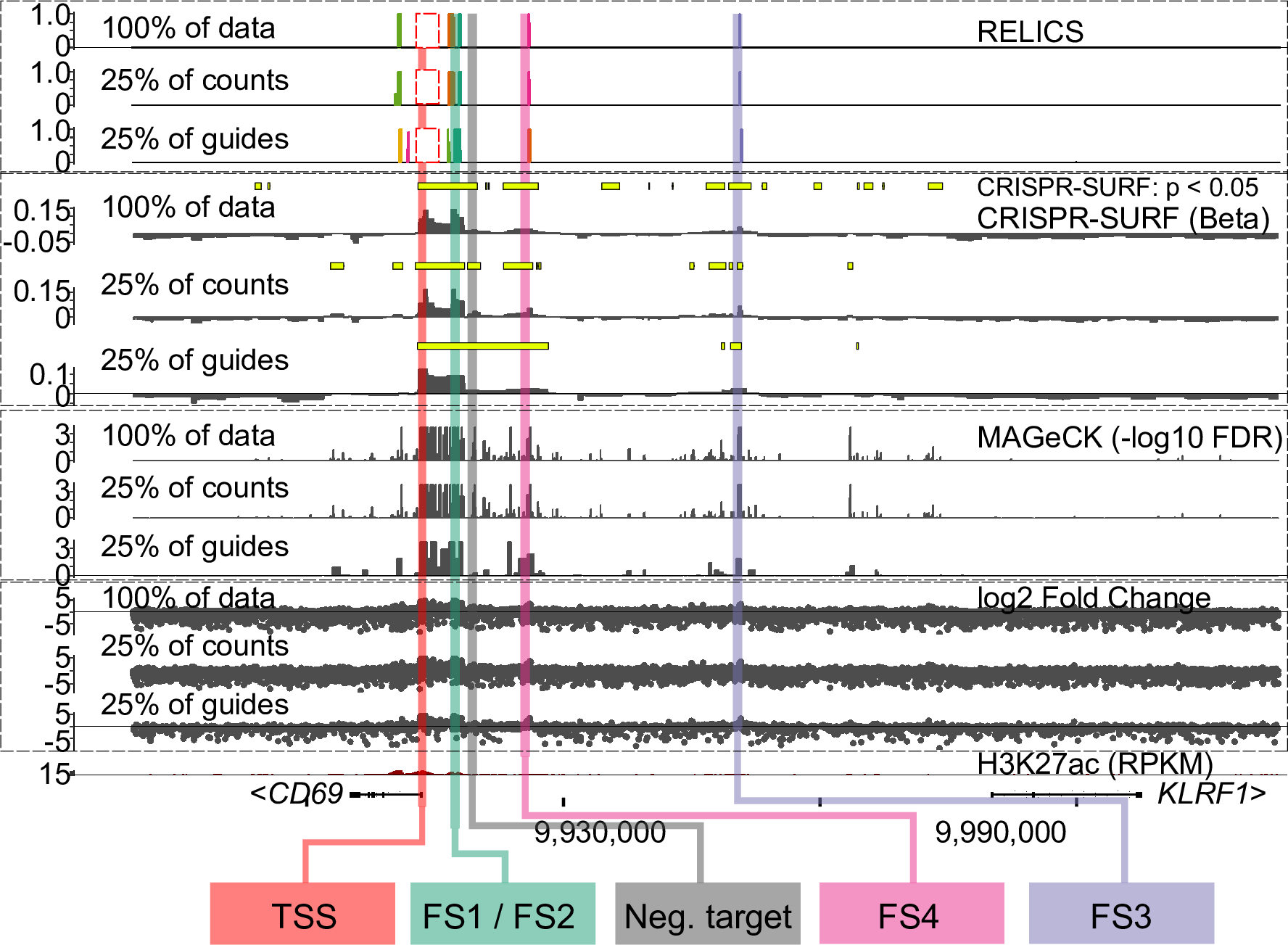

Supplement: S9 Fig — sgRNA counts were downsampled to 25% of all counts or sgRNA density was downsampled to 25%. Dashed blocks group results from different analysis methods together (RELICS, CRISPR-SURF, MAGeCK, log2 Fold Change). (TIF) [file pcbi.1008194.s009.tif]
