# Supplementary material for: Discovering functional sequences with RELICS, an analysis method for CRISPR screens
Source: PLoS Comput Biol. 2020 Sep 16;16(9):e1008194. doi: 10.1371/journal.pcbi.1008194 (PMC7521704; doi:10.1371/journal.pcbi.1008194)
Supplement: S5 Table — (DOCX) [file pcbi.1008194.s014.docx]

| **Variable** | **Description** |
| --- | --- |
| $J$ | Number of pools in screen |
| $K$ | Number of functional sequences |
| $L$ | Maximum length of functional sequence in genome segments |
| $M$ | Number of genome segments |
| $N$ | Number of sgRNAs |
| $\boldsymbol{y}$ | Observed sgRNA counts (matrix of dimension $N\times J$) |
| $\boldsymbol{\delta}$ | Functional sequence configuration (matrix of dimension $K\times M$). Each row, $\boldsymbol{\delta}_{\boldsymbol{k}}$ specifies the placement (length and position) of functional sequence $k$. A specific placement is denoted $\delta_{k}[m,l]$, where $m$ is the genome segment containing the start of the functional sequence and $l$ is the length of the functional sequence. |
| $\boldsymbol{\pi}$ | Probability a genome segment contains a specific functional sequence (matrix of dimension $K \times M$) |
| $\boldsymbol{\alpha}$ | Hyperparameters for sorting probability distribution (vector of length $J$) |
| $\boldsymbol{s}$ | sgRNA sorting probabilities (vector of length $N$) |
| $\boldsymbol{r}$ | Number of genome segments that an sgRNA overlaps that contain a functional sequence (vector of length $N$) |
| $\boldsymbol{p}$ | Probability genome segments contains any functional sequence (vector of length $M$) |
| $l$ | Length of a functional sequence |
| $g(n)$ | Mapping of sgRNAs to genome segments |
