# Supplementary material for: Discovering functional sequences with RELICS, an analysis method for CRISPR screens
Source: PLoS Comput Biol. 2020 Sep 16;16(9):e1008194. doi: 10.1371/journal.pcbi.1008194 (PMC7521704; doi:10.1371/journal.pcbi.1008194)
Supplement: Attachment — Submitted filename: Fiaux_et_al_rebuttal.docx [file pcbi.1008194.s015.docx]

We thank the reviewers for their time and constructive comments on our manuscript and we appreciate the opportunity to provide a revised version for consideration. In response to the reviewer comments we have made substantial improvements to the manuscript. The major improvements include:

1. We now evaluate the performance of RELICS and other analysis methods using a third-party simulation tool (Bodapati et al.) in addition to our own simulations. RELICS out-performs the other analysis methods (MAGeCK, BAGEL CRISPR-SURF) on both simulation datasets.
2. We now filter the guides used in all four experimental data sets using GuideScan with the score filtering recommended by Tycko et al. This filtering reduces the number of guides, but does not substantially alter RELICS’ results.
3. We perform additional experimental validations of 5 novel functional sequences predicted by RELICS on the *MYC* CRISPRi dataset by Fulco et al. We observe a substantial reduction in cell proliferation in 4 out of the 5 novel sequences and a small reduction in proliferation of 1 out of the five. The *MYC* results are now presented in Fig 4 in the main text.
4. We substantially revised the introduction and model description parts of the paper to make them less technical and make the paper accessible to a broader audience.

We have also made some additional improvements to the manuscript that were not specifically requested by reviewers:

5. One reviewer suggested swapping Fig 3 and Fig 4. Instead we have moved the old Figure 3 into the supplement (now part of Figure S5), because it was rather technical.
6. We corrected a bug in our precision calculations that was related to tied scores. As a result of the bug, the performance of MAGeCK was previously overestimated in some simulations.
7. We switched to using average precision as a performance metric instead of the area under the precision-recall curve. Average precision is a slightly better summary of a precision/recall curve, because it is not sensitive to the interpolation method that is used to connect points in the precision/recall curve.

We again would like to thank the reviewers for their constructive criticism. We address the specific concerns raised by each reviewer below.

Reviewer Comments:

**Reviewer #1:** The study entitled "Discovering functional sequences with RELICs, an analysis method for CRISPR screens" by Fiaux et al describes a novel method for analyzing pooled genetic screens. Overall, this study is clearly written and uses an innovative approach for screen analytics, which is distinct from previously described methods (BAGEL, MAGECK). The use of simulated and 'real' data is effective and strongly supports the power of this approach. In summary, I believe this study describes a useful tool that will be of broad utility to the field.

We thank the reviewer for the positive feedback.

**Reviewer #2:** In this manuscript, Fiaux et al. describe Regulatory Element Location Identification in CRISPR Screens (RELICS), a computational method to identify functional regulatory sequences from CRISPR tiling screens using sgRNA counts from multiple pools. To evaluate the performance of their method, the authors also developed a CRISPR simulation framework, CRSsim. They generated a total of 720 simulations using this framework by varying the strength of the guide, sequencing depth and the strength of selection. RELICS was tested on these simulated datasets and then compared to the predictions from BAGEL, MAGeCK and CRISPR-SURF. The authors then applied their method to existing CRISPRa/i tiling screens to show that RELICS predicts functional sequences predicted by other existing algorithms and also novel ones. They validated RELICS predicted functional sequences by using CRISPRa to overexpress CD69 assayed by flow cytometry.

Overall, RELICS could potentially help in discovering functional sequences using CRISPR screens but this manuscript needs to be supported with more evidence that this method is better than existing tools. The novelty of this method is not very clear in the current state of the manuscript.

As described below, we now perform additional validation experiments for sequences that are detected by RELICS but not detected by other analysis methods. We have also substantially revised the introduction and model description to better emphasize novel aspects of the RELICS model.

The lack of “ground truth” in most of these cases could be a problem. The authors clearly note in the discussion that prior knowledge of a positive control region is necessary to run this analysis and might be difficult to obtain.

We agree with the reviewer that a lack of ground truth dataset can make it difficult to evaluate the performance of a method, which is why we have included extensive simulations in our paper. As suggested by the reviewer below, we now also include simulations from the Bodapati et al. 2020. Even though these simulations were designed for gene-based screening methods such as MAGeCK, RELICS has the best overall performance on these simulations as well (Supplementary Fig. S4).

We now also include additional experimental validations for novel functional sequences detected by RELICS near the MYC locus, demonstrating that RELICS can detect functional sequences that are missed by other methods (Fig 4).

We do note in the paper that a limitation of RELICS is that does require the presence of a positive control sequence in the screen, however it is unusual and not advisable to perform a screen without positive controls. We now make note of this in the discussion:

“A limitation is that it might be difficult to apply RELICS to datasets that do not contain a known positive control region or that have a low number of sgRNAs overlapping positive control regions, although it is generally advisable to include positive controls in all screen designs.”

The existing tools used for comparison also do not seem relevant as elaborated below.

We thank the reviewer for pointing this out. Part of why we developed RELICS is because of a lack of tools to analyze tiling CRISPR screens. As more extensively addressed below, we compare RELICS against other tools, such as MAGeCK, which have previously been used to analyze these screens to draw attention to the fact that RELICS is the best choice for this type of analysis.

Major points

1. The authors mention that the simulation framework CRSsim generates “realistic datasets where the ground truth is known”. A figure comparing the data generated by this framework to some existing CRISPR datasets would strengthen this manuscript.

We agree with the reviewer that it is important to ensure that the simulated data sets resemble experimental data. Supplementary Figure 1 shows comparisons between a simulated dataset and a real dataset (from Fulco et al. 2016), and we have updated the figure to include a quantile-quantile plot comparing between the experimental and the simulated data (Supplementary Fig. S1c)

2. Base pair accuracy (BP) is defined as the fraction of significant bases identified which are true functional sequences. How are “true” functional sequences identified here? Also, in Figure 2, RELICS does predict functional sequences with higher BP accuracy than other methods especially for a guide with lower efficiency and weak selection strength but the BP accuracy does not get better for stronger guides or stronger selection strengths. Why is that the case?

We thank the reviewer for raising this point. In the revised manuscript we now explain that we were referring to the simulated functional sequences as the ‘true’ FS.:

“We quantified the resolution of predicted functional sequences with basepair (BP) accuracy—the total number of significant genomic bases that overlap with true, simulated, functional sequences. For each simulated functional sequence, we quantified the fraction of base pairs that were called significant and overlapped a simulated functional sequence.”

We also now include a section in ‘Materials and Methods’ that describes why there is a maximum attainable BP accuracy and why the BP accuracy is low in the CRSsim simulations and higher BP in the Bodapati et al. simulations:

“The BP accuracy for the CRSSim simulations was not very high for all methods. This is due to the large area of effect (1000bp) and the small functional sequence size (50bp) used in these simulations. This area of effect is typical of CRISPRi and CRISPRa. RELICS used a genome-segment size of 100bp segments, so the best possible BP accuracy for a functional sequence that is wholly contained with a genome segment is 50%. In the case where a true FS (50bp) is split between two segments and RELICS labels correctly labels both segments as FSs, the BP accuracy is only 25%. The Bodapati simulations do not include an area of effect (i.e. the area of effect is 1bp), and RELICS used 20bp genome segments. This simulation setup allows for finer-resolution predictions, resulting in higher BP accuracy (Supplementary Fig. S4).”

3. The authors use four other methods to compare RELICS to. The log-fold change is just a simple transformation of the data and not really a “method”. Likewise, BAGEL and MAGeCK are approaches to find essential genes, not identify functional elements. These comparisons should be excluded and the only relevant comparison to CRISPR-SURF should be included in this manuscript.

We thank the reviewer for pointing this out, and we have updated the manuscript to clarify that we do not actually compare against log fold change. However, we do include the log fold change tracks in the figures for the 4 data sets we analyze because we believe that it provides a useful view of the raw data.

We also agree with the reviewer that both BAGEL and MAGeCK are designed for gene-based CRISPR screens. However, we wish to include a comparison to MAGeCK because it is very popular for CRISPR screen analysis, and it has been previously used to analyze a tiling non-coding CRISPR screen (Diao et al. 2017). We feel that it is important to demonstrate that RELICS is a better choice for tiling screens. We have included BAGEL, because it is similar to MAGeCK, but is a supervised method. We wish to demonstrate that the better performance of RELICS is not simply due to a difference between supervised vs. unsupervised methods. We now clarify the reasons for comparing against MAGeCK and BAGEL in the manuscript:

“While both MAGeCK and BAGEL are designed for gene-based screens, we compare against them because MAGeCK is the most popular analysis method for CRISPR screens and has previously been used to analyze tiling CRISPR screens [11]. We use BAGEL to provide a comparison to another method that utilizes training data to estimate model parameters.”

4. The authors also show that their method is better than BAGEL, MAGeCK and CRISPR-SURF using precision-recall AUC and BP accuracy on the simulated datasets. These metrics should also be used to demonstrate that RELICS is better than the other methods using the published data sets.

We thank the reviewer for this comment and agree that it would be great to be able to compute precision, recall and BP accuracy on the published data sets. Unfortunately, it is very challenging to compute these metrics on experimental data because the ground truth for most targeted sites is not known.

- For BP accuracy we need to know the exact location of the FS (i.e. which bases are functional), which is not known.
- Precision can be estimated using experimental validations performed on a set of FSs predicted by a method, however it is difficult to compare precision estimates for different methods without systematically testing every positive prediction made by every method being compared. Computing precision using the FSs predicted just by RELICS (e.g. those we perform validation experiments for at the MYC locus) is potentially biased because these elements were selected because they were predicted by RELICS and not the other methods.
- Recall cannot be estimated without knowing the true number of FSs within the screened region.

For these reasons we prefer to report precision, recall, and BP accuracy on the simulated datasets where the ground truth is known, and to describe the experimental validations on real datasets, without comparing the precision directly.

Only a single guide is used to validate the “negative sequence” prediction that was made by MAGeCK and is in a significant portion predicted by CRISPR-SURF but not RELICS. The Rule Set 2 score of this guide is 0.39 which makes it an inefficient guide. The authors do note in the legend of Figure 4 that “Targeting sgRNAs were chosen for their high predicted efficiency”. Based on what criteria were the targeting sgRNAs picked? Also, when a single guide is used for validation, it is difficult to determine if CD69 activation was not observed due to the inefficiency of the guide or the region did not indeed have a functional sequence. The authors do validate most other regions with 2 sgRNAs but why was this region only targeted with one guide?

We chose sgRNAs from all of the possible sgRNAs in the target region prioritizing those without predicted off-target effects, and then high predicted efficiencies relative to the other guides with good off-target scores. We used the Doench on-target, CFD and Hsu scores generated by FlashFry. We designed two guides to target the “negative sequence.”, but unfortunately we were not able to clone the sgRNA with the highest predicted efficiency.  The guide that we used had the highest efficiency (of those with high Hsu off-target scores), with FlashFry reported scores of Doench on-target: 0.323, Doench CFD: 0.224, Hsu: 99.35.

We have modified the Figure legend to indicate that we chose guides that had high efficiency relative to other possible guides:

“Targeting sgRNAs were chosen for their high specificity and high predicted efficiency (relative to possible sgRNAs in the region) and in some cases are adjacent to the predicted FS rather than within the FS.”

We now also describe the guide selection criteria in the Methods:

“We used FlashFry [31] to design two sgRNAs per target site. FlashFry calculates guide efficiency and off-target effects for each guide by reporting the Doench 2014 [38], Doench 2016 [26], and Hsu 2013 scores [28]. For each of the sites we selected guides with low estimated off-target effects and high estimated efficiency relative to the other possible guides in the region.”

In the new version of the manuscript, we perform filtering of all sgRNAs using GuideScan prior to running all of the analysis tools. After GuideScan filtering the “negative sequence” is no longer predicted by MAGeCK, although it still overlaps with a large significant region predicted by CRISPR-SURF. We still include the results from targeting this sequence, but have removed the claim that this method is a false-positive prediction by the other methods. The new text is:

“Finally, targeting a “negative sequence” that was not predicted by RELICS, but is on the edge of a large significant region reported by CRISPR-SURF did not change *CD69* expression.”

6. To support their claim of RELICS identifying new regions, the authors should validate the 5 regions identified around the MYC locus that have not been reported previously. This would help in proving the novelty of this method.

Thank you for this suggestion. We have performed the validation experiments and feel that the results have substantially improved the novelty of the manuscript. With GuideScan filtering, RELICS now predicts 6 novel functional sequences for the *MYC* locus instead of 5. We performed validation experiments for 5 of the sequences (FS9, FS10, FS12, FS14, FS15). The results from these experiments are shown in Figure 4, which is now a main text figure. We have added the following description to the Results:

“Interestingly, RELICS also identified 6 regions that have not been previously reported and that neither CRISPR-SURF nor MAGeCK detected. To test whether these sequences are functional we targeted 5 of them with CRISPRi. As a positive control we targeted a previously-detected functional sequence reported by Fulco et al. (referred to as e2), and as negative controls we used dCas9:KRAB alone or dCas9:KRAB with an sgRNA targeting a safe harbor sequence on chromosome 8 with no known function. Of the 5 FSs we tested, 4/5 showed a substantial decrease in proliferation and 1/5 (FS10) showed a small decrease in proliferation with at least one of the targeting sgRNAs (Fig. 4b). This confirms that RELICS discovered additional functional sequences that are missed by other methods.”

The new figure follows:

**Figure 4** | Analysis of a *MYC* CRISPRi cellular proliferation screen published by Fulco et al. 2016. **(a)** Output of RELICS and other analysis methods. Each FS predicted by RELICS is given a different color and the labels are arranged by genomic position. **(b)** Experimental validations. Each validation experiment is a cellular proliferation assay, in which the percent of GFP-positive cells (i.e. those that received the sgRNA) are measured at day 0, day 6 and day 14. As negative controls we used dCas9:KRAB only (no sgRNA) as well as sgRNAs targeting a ‘safe harbor’ non-functional region on chromosome 8. As positive controls we used sgRNAs targeting a known regulatory region (e2, identified by Fulco et al., corresponding to FS2 and FS3). Each functional sequence was targeted with either one, two, or three different sgRNAs with two replicates each. sgRNAs that resulted in a significant reduction in % GFP compared to negative controls (dCas9 only, Neg. Ctrl.) at day 14 have an asterisk (Student’s one-sided t-test, p < 0.05).

7. RELICS was easy to use and the instructions were very clear on GitHub. Well documented tools are hard to find and the authors have done a very good job on explaining the various parameters that can be used to run this tool.

Thank you, we have strived to make RELICS well-documented and easy to use for other researchers.

8. Bodapati et al., 2020 describes a simulation framework for CRISPR screens and the use of similar hyperparameters as described in this paper. It might be useful to compare CRSsim to their framework and also apply RELICS to datasets simulated by their framework.

We thank the reviewer for pointing us to the simulation tool by Bodapati et al. While the Bodapati simulation tool is specifically designed to simulate gene-based CRISPR screens we were able to adapt its output to make it resemble a tiling screen. We present the results from the Bodapati et al. simulations in Figure S4, and mention these simulations in the Results:

“We also simulated data using a tool recently developed by Bodapati et al. [27]. Using the data from these simulations RELICS again outperformed all other methods in AP, precision, recall and BP accuracy (see methods, Supplementary Fig. S4).”

And we describe how we performed the Bodapati et al. simulations in the Methods:

**“Bodapati et al. simulations.** We downloaded the Bodapati et al. simulation tool from GitHub (<https://github.com/sbodapati/CRISPR_Benchmarking_Algorithms>). We varied guide efficiency (low = 0.4 / high = 0.8), the number of guides per gene (low = 5 / high = 20) and the sequencing depth (low = 100 / medium = 200 / high = 400). We performed 15 simulations for various combinations of guide efficiency, guides per gene, and sequencing depth. This resulted in 12 scenarios and a total of 180 simulations, each with ~8400 sgRNAs and 8 functional sequences. For simulations with 5 guides per gene we simulated a total of 36 essential genes. Of those, 28 were used as training data for FS0, the remaining 8 were used as functional sequences which are to be detected. In simulations with 20 guides per gene we simulated 15 essential genes of which 7 were used as a known positive control functional sequence (FS0) and the remaining 8 were treated as unknown functional sequences. This resulted in ~150 guides as FS0 training which is similar to the datasets we analyzed in this paper. To keep the same number of guides across the scenarios we simulated 1653 or 407 non-essential genes with either 5 guides or 20 guides per gene respectively.

Since the Bodapati et al. simulation tool was originally designed for gene-based screens we adapted the data to resemble a tiling CRISPR screen. We first randomized the order of the non-essential genes and the genes used as functional sequences while holding out the genes used for FS0. Next, we mapped all genes to genomic coordinates with guides spaced 6 bp apart (6 bp was used because that was the average tiling density of Fulco et al. 2016). All genes used for FS0 training were mapped to the middle of the tiling region to mimic the gene of interest.”

Minor points

1. Typo in Line 152, it should be “functional sequence probabilities”.

We have now corrected this

2. In line 157, the authors mention that RELICS predictions on the simulated datasets were compared to “log-fold change”, but this comparison has neither been demonstrated in the main or supplementary figures nor has been further discussed in the text.

We thank the reviewer for pointing this out. We do not compare against log-fold change and we have removed this statement from the text.

3. In line 190, Figure 3(a, b) are referenced but do not directly correspond to the text. Figure 3(c, d) are referenced after Figure 4. It might be helpful to re-order the figures to go with the flow of the text.

We agree that Fig 3 did not fit with the flow the text. Since the figure is rather technical, we decided to place it in the supplement instead (as Figure S5).

In Figure 5b., indicate what the two lines for GLOD5 sgRNA1, GLOD5 sgRNA2 and Negative control indicate? Are they replicates?

We have clarified in the Figure text that the different lines represent different replicates.

5. Supplementary Figure 3 & 4 indicate the method used as “RELICS 2” but other figures say “RELICS ''. Please explain the difference between these methods.

RELICS2 was an internal name for this version of the software. We have corrected this and we have now changed all of the labels to RELICS

6. Please include why and how sgRNAs were filtered to run CRISPR-SURF on the experimental datasets?

We have updated the CRISPR-SURF section of the methods to describe that it performs its own filtering. We run the CRISPR-SURF commands as specified in the supplemental material for the CRISPR-SURF method. The updated description is as follows:

**Running CRISPR-SURF.** CRISPR-SURF (downloaded 02/13/2019 from GitHub) was run using the procedure specified in the supplemental materials of Hsu et al. 2018. CRISPR-SURF analyzes count data in two steps. The first step filters out guides of low quality and computes log2 fold change (Command ‘SURF_count’). The second step performs the deconvolution (Command ‘SURF_ deconvolution) to identify functional sequences. During the first step, all guides which have a count less than 50 in any of the pools get removed. While this works with data sets that have been sequenced deeply, this can become an issue when the sequencing depth is not as high. For this reason, to run CRISPR-SURF on the simulated datasets, we set the guide count filter to 15 instead of 50. For the experimental datasets we used the default guide count filter of 50. CRISPR-SURF accepts positive control as input, which can improve the output. We provided CRISPR-SURF with the same positive controls that RELICS used for training. The SURF_count command was run as follows:

docker run -v $Path_to_file/:$Path_to_file -w $Path_to_file pinellolab/crisprsurf SURF_count -f CRISPR_SURF_count.csv -nuclease cas9 -pert crispri

The SURF_deconvolution command was run as follows:

docker run -v $Path_to_file/:$Path_to_file -w $Path_to_file pinellolab/crisprsurf SURF_deconvolution -f CRISPR_SURF_Input.csv -pert crispri

7. Another analysis that might be worth adding would be to filter out promiscuous guides from the tiling libraries as shown by Tycko et al., 2019 and re-assessing the performance of RELICS.

We thank the reviewer for pointing this out to us. We have now run GuideScan and performed the same filtering described by Tycko et al. on the four experimental data sets prior to running all analyses. All of the described results are now based on the filtered data sets. Overall, the results for the methods have not changed substantially. The biggest differences are that (i) the “negative sequence” for CD69 is no longer predicted by MAGeCK, (ii) the GLOD5 functional sequence near GATA1 is no longer predicted by CRISPR-SURF or MAGeCK (it was never predicted by RELICS), and (iii) RELICS now predicts an additional functional sequence for the *MYC* locus.

**Reviewer #3:** The paper by Fiaux et al. introduces a Bayesian hierarchical model, which the authors termed RELICS, designed to help identifying functional sequences using CRISPR screens. The model performs best when offered data that contain positive controls (known functional sequences) and as such uses a semi-supervised approach. To test the performance of their model, the authors also developed an open source simulation tool, CRSsim, which should be useful for cross-validation of performances of different models. The RELICS method seems interesting and may embody a considerable advance as it outperformed existing models in the analyses shown. The manuscript is well-written and the data are aesthetically well present.

Major point:

The RELICS methodology is not well explained and confusingly presented. Although most of the information can be found in the manuscript, most readers will miss it because it takes a while to find. In some sections the presentation is a bit too technical and as such makes for a tedious read. To make this manuscript more accessible to a broader authorship, the authors are encouraged to walk the reader through the key design steps of RELICS in plain language, to reduce jargon and where needed explain terminology on first use. In its current form, readers will also find it difficult to fully appreciate the strengths of the methodology and to independently validate that comparisons to existing models were undertaken in a way that did not favor RELICS.

We thank the reviewer for this valuable input. We have extensively revised both the introduction of the manuscript and the “RELICS model” portion of the results. We have made the language less technical and try to clearly convey the main challenges and design features of RELICS to the reader. We feel that these changes have substantially improved the readability of the manuscript and should make it accessible to a broader audience.

Minor points:

Figure 1a: The ‘area of effect’ depictions in this schematic are indicating identical spans for each guide. In reality, guides that target overlapping functional sequences would be expected to often have the boundaries of their ‘areas of effect’ coincide.

We think that this is a misconception by the reviewer due to our confusing description of area of effect in the previous version of the manuscript. The schematic is accurate because RELICS does assume that the area of effect is the same for all sgRNAs and that the area of effect does not change depending on the presence or absence of a functional sequence. To make this less confusing, we have updated our description of the RELICS model extensively including descriptions of area of effect.

In the introduction when describing analysis challenges we now describe the ‘area of effect’ this way:

“The analysis of tiling CRISPR screens poses numerous challenges including […] the ‘area of effect’ of genomic perturbations, which often extends well beyond the genomic location targeted by the sgRNA (e.g. in CRISPRa or CRISPRi screens activating or silencing epigenetic modifications can spread over 1kb or more from target sites [15])”

In the RELICS model section of the results we now describe the ‘area of effect’ and its relationship to genome segments and functional sequences as follows:

“It is also important to consider the genome locations of both functional sequences and sgRNA target sites as well as the area of effect for each sgRNA. The area of effect of CRISPR perturbations means that a single sgRNA can potentially affect multiple functional sequences in the vicinity of the target site. Similarly, a single functional sequence can be perturbed by multiple sgRNAs with nearby target sites. To model the spatial organization of functional sequences and sgRNA-directed perturbations, RELICS divides the sequence targeted by the screen into small windows called genome segments. Each genome segment is then associated with all sgRNAs that are predicted to affect that genome segment, based on their overlap with the sgRNA’s area of effect (Fig. 1c).”

Figure 1b and c: The authors chose to depict guides in different pools with the same colors, which I presume does not indicate that indeed the guides used in the different pools were identical? This is confusing and should be changed unless I misunderstood the methodology.

The guides in the different pools are identical, but they have different observed counts in the different pools (i.e. they are present in a different fraction of cells). All of the guides are first delivered to cells in an input pool of cells and then the cells are sorted or a selection is performed to create the other pools of cells. We have added the following paragraph to the Introduction to try to make this more clear to the reader.

“Following genomic perturbation, the cells are subsequently sorted into pools based on a cellular phenotype (e.g. high vs. low gene expression, survival, proliferation), and the distributions of sequenced sgRNAs are compared across pools to identify functional genomic sequences that affect the cellular phenotype used for sorting. For example, sgRNAs that disrupt activating regulatory sequences would result in reduced target gene expression and thus be enriched in pools selected for low target gene expression.”

P18, Lines 335 -369: It seems that positive controls (i.e., known functional sequences) were specified only for RELICS, not any of the other platforms it performance was compared against. Since the authors mentioned that RELICS may be difficult to apply to datasets without an identified positive control (line 265), would the lack of a positive control have disadvantaged the performance of the other platforms, and if so, to which extent would this have compromised their performance when compared with RELICS?

We thank the reviewer for this comment and now make it clear in the manuscript that in addition to RELICS, BAGEL and CRISPR-SURF were provided with same positive controls as RELICS (this is described in the Running CRISPR-SURF and Running BAGEL sections). We agree with the reviewer, that a lack of positive controls would make it difficult to apply RELICS, but note that well-designed screens should contain positive controls. We now note this in the Discussion:

“A limitation is that it might be difficult to apply RELICS to datasets that do not contain a known positive control region or that have a low number of sgRNAs overlapping positive control regions, although it is generally advisable to include positive controls in all screen designs.”

P23, Lines 487-488: Note that the font is changing in unexpected ways in this section.

We thank the reviewer for pointing this out and have updated it in the current version of the manuscript.

Overall, this reviewer is excited about this paper but strongly advises the authors to change the presentation such that the key design features and steps distinguishing RELICS from existing methodologies are explained early on.

We thank the reviewer for their comments, which we feel have improved the manuscript substantially. In particular, we thank the reviewer for their suggestion to revise the manuscript to make it easier to understand and to broaden its audience.
